# Impact of Neuroendocrine Neoplasm-Specific Systemic Treatments on Somatostatin Receptors Expression and Function in Neuroendocrine Tumor Cells

**DOI:** 10.3390/cancers18091368

**Published:** 2026-04-25

**Authors:** Christof Däubler, Clara Böttcher, Laura-Sophie Landwehr, Philipp E. Hartrampf, Alexander Meining, Rudolf A. Werner, Yingjun Zhi, Otilia Kimpel, Simon Kloock, Ulrich Dischinger, Alexander Weich, Dorothee Rogoll

**Affiliations:** 1 Department of Internal Medicine II, Gastroenterology, University Hospital Würzburg, 97080 Würzburg, Germany; daeubler_c1@ukw.de (C.D.);; 2 Department of Internal Medicine I, Endocrinology, University Hospital Würzburg, 97080 Würzburg, Germany; 3 Department of Nuclear Medicine, University Hospital Würzburg, 97080 Würzburg, Germany; 4 Department of Nuclear Medicine, LMU University Hospital, LMU Munich, 81377 Munich, Germany; 5 Department of Otorhinolaryngology, Head and Neck Surgery, University Hospital Würzburg, 97080 Würzburg, Germany

**Keywords:** neuroendocrine neoplasm, somatostatin receptor, systemic therapeutics, radionuclide uptake

## Abstract

**Background**: Somatostatin receptors (SSTRs) are pivotal diagnostic and therapeutic targets in well-differentiated neuroendocrine neoplasms (NENs). SSTR-directed treatment strategies rely on sufficient SSTR2 expression. Thus, receptor loss during dedifferentiation limits therapeutic efficacy. Preclinical data suggest pharmacologic modulation of SSTRs’ expression. However, there are no robust data on the effect of NEN-specific systemic treatments on SSTRs’ expression and function. **Methods**: We systematically evaluated the effects of six systemic agents commonly used in NEN therapy—isplatin, etoposide, 5-fluorouracil (5-FU), streptozotocin (STZ), temozolomide (TMZ), and everolimus—on SSTR2 and SSTR5 expression, as well as on uptake of ^68^Ga-DOTATOC, in BON-1 and QGP-1 cells, as well as the MS-18 cell line. Analyses included qRT-PCR, Western blotting, immunohistochemistry, and radiopeptide uptake assays. **Results**: Systemic agents modulated SSTR expression and radioligand uptake in a drug- and cell line-dependent manner. Etoposide consistently upregulated SSTR2 expression and significantly increased radioligand uptake across all three cell lines. TMZ enhanced SSTR2 expression and uptake in BON-1 cells, but reduced uptake in QGP-1 and MS-18 cells. In contrast, 5-FU, STZ, cisplatin, and everolimus showed heterogeneous, compound- and cell line-specific effects on SSTR2 expression and ^68^Ga-DOTATOC uptake, including both up- and downregulation depending on the model. **Conclusions**: All agents under investigation affect SSTR expression in vitro, while etoposide is identified as the most consistent enhancer of SSTR2’s expression and function across cellular NEN models. Our findings highlight both the potential and the risks of systemic therapy-induced receptor modulation and therefore support further investigation of treatment sequencing strategies to optimize SSTR-targeted approaches. However, further studies are required to translate these observations to a clinical setting.

## Simple Summary

Somatostatin receptors (SSTRs), particularly SSTR2, are key targets for imaging and therapy in well-differentiated neuroendocrine neoplasms (NENs). However, their expression is dynamic and may be altered during disease progression or under systemic treatment, potentially affecting the efficacy of SSTR-directed approaches. In this study, we systematically evaluated the impact of commonly used systemic therapies on SSTRs’ expression and function in three NEN cell lines (BON-1, QGP-1, MS-18). Cisplatin, etoposide, 5-fluorouracil, streptozotocin, temozolomide, and everolimus were assessed regarding SSTR2/SSTR5 expression and ^68^Ga-DOTATOC uptake. Effects were highly drug- and cell line-dependent. Etoposide consistently upregulated SSTR2 and significantly increased radioligand uptake across all models, identifying it as the most robust enhancer. In contrast, temozolomide showed heterogeneous effects, increasing uptake in SSTR-low but reducing it in SSTR-high models. Other agents demonstrated variable and partly opposing effects. Overall, these findings highlight the importance of treatment selection and sequencing for optimizing SSTR-targeted strategies.

## 1. Introduction

Neuroendocrine neoplasms (NENs) are a heterogeneous group of tumors that can arise in nearly any organ system, most commonly in the gastrointestinal tract (48–51%), lungs (25%), and pancreas (9%) [1,2]. Their incidence has steadily increased over recent decades [2]. Depending on their degree of differentiation, NENs exhibit variable neuronal and endocrine features [1]. Well-differentiated NENs often retain tissue-specific traits, including somatostatin receptor (SSTR) expression and hormone secretion [1,2,3]. In well-differentiated neuroendocrine tumors (NETs), the preservation of SSTR expression—particularly of SSTR2—provides the molecular basis for receptor-targeted diagnostics and therapy and is associated with a more favorable prognosis [4]. Conversely, poorly differentiated neuroendocrine carcinomas (NECs), or NETs that undergo dedifferentiation during disease progression, often lose SSTR expression [5,6,7], which is associated with a substantially worse prognosis [4,8,9,10].

SSTR2 and, to a lesser extent, SSTR5 are the most important diagnostic and therapeutic targets in NENs, not only enabling diagnostic imaging (e.g., DOTA-PET/CT) and peptide receptor radionuclide therapy (PRRT), but cold somatostatin analogs (SSAs) also exert direct biological effects through SSTR-mediated signaling. These include inhibition of hormone secretion, suppression of proliferation, reduced tumor cell migration, and induction of apoptosis [11,12]. The clinical benefit of SSAs has been demonstrated in the PROMID and the CLARINET trials, both of which showed significant improvements in progression-free survival (PFS) in patients with metastatic gastroenteropancreatic (GEP) NETs treated with octreotide or lanreotide [13,14]. Furthermore, the Phase III NETTER-1 trial demonstrated a significant advantage in PFS for PRRT in G1/G2 NETs, which were progressive under SSA treatment [15,16]. Importantly, the efficacy of SSTR-directed therapy strongly depends on the level of receptor expression, particularly SSTR2 [17,18].

Alternative systemic treatments for NENs comprise chemotherapy (e.g., temozolomide (TMZ)/capecitabine, streptozotocin (STZ)/5-FU, etoposide, cisplatin), and targeted agents (such as everolimus) are applied when progression occurs under SSTR-directed therapy or the tumor shows insufficient expression [19,20].

These systemic treatments may exert effects on SSTR expression and consequently influence the efficacy of SSTR-directed diagnostic or therapeutic strategies [21,22]. In radiation oncology, for example, chemotherapeutics are often used as radiosensitizers to increase radiotherapy effectiveness [23]. The first in vivo data could show the potential of combining TMZ with PRRT in a murine SSTR-expressing small cell lung cancer model [24]. However, there are only sparse data on the combination of systemic therapies with SSTR-directed diagnostic or therapeutic approaches in NENs. Two in vitro studies have examined the effects of a few selected systemic agents on SSTR expression and radionuclide uptake in NET cells. Shah et al. (2021) reported that combining (177)Lu-DOTA-Octreotate with TMZ, 5-FU, or STZ increased radionuclide uptake in the pancreatic cell line BON-1 and enhanced anti-proliferative effects compared with PRRT alone [21]. Similarly, Jin et al. (2019) demonstrated that 5-FU, alone or combined with epigenetic modulators, increased SSTR2 expression and ^68^Ga-DOTATOC uptake in BON-1 and QGP-1 cells [25]. Despite these insights, both studies are limited by the small set of cell lines, which do not contain a patient-derived cell line with high SSTR expression for studying receptors’ expression and function. Moreover, only a few selected substances have been tested, instead of a structured comparative evaluation of guideline-relevant systemic therapies across multiple NET cell lines with preserved SSTR expression being performed. For improving SSTR-directed diagnostic and therapeutic strategies as well as for preventing possible interactions with other systemic agents, which may interfere with SSTR expression and jeopardize treatment success, comprehensive data on the effect of NEN-specific systemic agents on SSTR expression and function are required to form the basis of in vivo studies and clinical trials.

Therefore, the present study systematically investigated the effects of a multitude of guideline-relevant systemic agents—cisplatin, etoposide, 5-FU, streptozotocin, temozolomide, and everolimus—on SSTR2 and SSTR5 expression and on ^68^Ga-DOTATOC uptake in three established NET cell lines (BON-1, QGP-1, and MS-18) [26,27,28]. This design allows a more comprehensive assessment of potential modulation of SSTRs’ expression and function across different cellular contexts, opening avenues for optimizing SSTR-directed diagnostic or therapeutic approaches like SSTR-directed imaging, PRRT, or treatment with cold somatostatin analogs.

## 2. Material and Methods

### 2.1. Cell Culture

The pancreatic BON-1 and QGP-1 human NET cell lines originated as a generous gift from Prof. C. Grötzinger from the Department of Hepatology and Gastroenterology, Charité-Universitätsmedizin Berlin, Germany. The MS-18 cell line, derived from a metastasized NEC of the rectum, is a primary cell line described elsewhere [28]. To ensure the absence of mycoplasma contamination, all cell lines were verified using Venor GeM OneStep (Cat#11-8025, Minerva biolabs, Berlin, Germany). In culture, QGP-1 cells were maintained in RPMI-1640 medium (Cat#21875-034, Gibco, Carlsbad, CA, USA), while BON-1 cells were cultured in Dulbecco’s Modified Eagle’s Medium + Nutrient Mixture F12 Ham (Cat#D6546 and N4888, Sigma Aldrich, Darmstadt, Germany). Both media were supplemented with fetal bovine serum (Cat#S06115, FBS; 10% *v*/*v*; FBS Superior, Biochrom, Berlin, Germany) and penicillin/streptomycin (Cat#15140-122, Pen/Strep 1 × 10^5^ U/mL, Thermo Fisher Scientific, Waltham, MA, USA). The MS-18 cells were cultured in Dulbecco’s Modified Eagle’s Medium/F12 medium (Cat#11330-032, Gibco, Carlsbad, CA, USA), with supplementation of Nu-Serum (Cat#355100, 3% *v*/*v*; Corning Inc., Corning, NY, USA) and 1% insulin–transferrin–selenium (Cat#41400-045, Gibco, Carlsbad, CA, USA). Cell media replenishment occurred every 3–4 days, with a maximum of 30 passages. The drugs employed in this study included everolimus and temozolomide (TMZ) (Cat#S1120 and S1237, Selleckchem, Berlin, Germany), 5-fluorouracil (5-FU), etoposide (Cat#: F6627 and E1383, Sigma-Aldrich, Darmstadt, Germany), cisplatin (Cipla, Antwerpen, Belgium), and streptozotocin (STZ) (Cat# ALX-380-010-M100, Enzo Life, Lörrach, Germany).

The incubation times with the compounds were based on EC50 values and observed cell survival. For BON-1 and QGP cells, the following treatment conditions were applied: Cisplatin at 100 µM for 48 h, etoposide at 300 µM for 24 h, streptozotocin at 2.5 mM for 24 h, 5-FU at 750 µM for 48 h, temozolomide at 100 µM for 96 h, and everolimus at 100 nM for 48 h. For MS-18 cells, the concentrations were adjusted due to lower cell numbers to ensure sufficient viable cells for downstream experiments. The following concentrations were used: Cisplatin at 10 µM for 48 h, etoposide at 25 µM for 24 h, streptozotocin at 0.75 mM for 24 h, 5-FU at 150 µM for 48 h, temozolomide at 100 µM for 96 h, and everolimus at 100 nM for 48 h. The selection of concentrations was based on previously published data [28].

### 2.2. RNA Isolation and Quantitative RT-PCR

Total RNA was extracted from cell cultures using NucleoSpin RNA (Cat#740955, Machery-Nagel, Düren, Germany), and cDNA synthesis was performed by reverse-transcribing 1 µg of total RNA with iScript (Cat#1708891, BioRad, Hercules, CA, USA), following the manufacturer’s instructions. Prevalidated primers or TaqMan gene expression assays, along with endogenous controls, were procured from Thermo Fisher Scientific/Applied Biosystems (Foster City, CA, USA). Further details can be found in Supplementary Table S1. Samples in triplicate underwent qRT-PCR using the Absolute QPCR Mix (Cat# AB1132/B, Thermo Fisher Scientific, Waltham, MA, USA) and were detected using an AB ViiATM7 Real-time PCR System (Life Technologies, Carlsbad, CA, USA). Expression levels were normalized to the housekeeping gene HPRT1 using the 2^−∆∆Ct^ method. All experiments were performed with six independent biological replicates.

### 2.3. Western Blotting

The Western blot analysis was conducted using this briefly described method: cells were seeded at a density of 5 × 10^5^ cells per well in 6-well plates (Cat#657160, Greiner, Bio-one, Frickenhausen, Germany), washed with 1× PBS (Cat#AC-BS-0002, Anprotec, Bruckberg, Germany), and then harvested in a lysis buffer (Cat#89900, Ripa buffer, Thermo Scientific, Foster City, CA, USA) supplemented with a protease inhibitor cocktail (Cat#78429, Thermo Scientific, Foster City, CA, USA). The protein content was determined enzymatically using the BCA protein assay (Cat#23227, Thermo Scientific, Foster City, CA, USA), and 30 µg of the samples was loaded onto SDS-polyacrylamide gels (Cat#1610158, 10% polyacrylamide; BioRad) and separated by electrophoresis. For immunoblotting, proteins were transferred onto Immobilon-P PVDF transfer membranes (Cat#IPVH00010, Merck, Millipore, Burlington, USA). The membrane was blocked with 5% nonfat dried milk (Cat#A0830, Applichem, Darmstadt, Germany) in Tris-buffered saline and incubated with the primary antibodies and horseradish peroxidase (HRP)-conjugated secondary antibodies (details provided in Supplementary Table S1). Visualization was achieved using a chemiluminescence detection system (Cat#1705061, 1610375, BioRad; ECL Clarity Western ECL Substrate, Precision Plus Protein Standards). Band intensities were quantified using GelAnalyzer 23.1.1 software [29]. Due to known antibody characteristics, multiple bands were observed; quantification was based on bands at the expected molecular weight. SSTR signal intensities were initially normalized to the β-actin loading control to account for differences in sample loading, and then further normalized to the medium-treated control within each experiment to enable comparison across treatment groups. Three independent biological replicates were conducted, and the normalized data were used for statistical analysis.

### 2.4. ^68^Ga-DOTATOC Uptake

Cellular uptake of ^68^Ga-DOTATOC was assessed in BON-1, QGP-1, and MS-18 cells cultured in 24-well plates (Cat#665180, Greiner, Bio-One). After pre-treatment with the respective compounds for the indicated time, culture supernatants were removed and cells were incubated with 500 µL PBS/5% BSA for 30 min. ^68^Ga-DOTATOC was prepared at a total activity of approximately 11 MBq in 8 mL PBS, and 50 µL of this solution was added per well in triplicate. For each cell line, three reference aliquots were included. Following incubation for 60 min at 37 °C, uptake was stopped by placing the plates on ice for 2 min. Thereafter, the supernatant was removed, and the cells were washed with PBS, detached by trypsinization, collected in microcentrifuge tubes, and washed twice with PBS. Cell-associated radioactivity was measured using a gamma counter (Wizard2 2480 gamma counter, Perkin Elmar, Waltham, MA, USA). Subsequently, cells were counted, and uptake values were normalized to 10^6^ cells. The normalized uptake was then related to the corresponding reference samples and expressed as percentage uptake (% uptake). All measurements were performed in triplicate and independently repeated at least three times.

### 2.5. Immunohistochemistry

Immunohistochemistry status and simultaneous localization of specific proteins in BON-1, QGP-1, and MS-18 cells were assessed using cytospins with a total of 2 × 10^5^ cells per spin. Therefore, cells were grown in 6-well plates (Cat#657160, Greiner-Bio-One) and supplemented with or without different drugs. Antibodies used in this study were applied using the super-sensitive streptavidin–peroxidase anti-peroxidase technique (Cat#QP900-9LE, Biogenex, Fremont, CA, USA). Details on antibodies are also included in Supplementary Table S1. Microphotographs were taken with EVOS M5000 equipped with an EVOS Objective (40×). Staining intensity was assessed semi-quantitatively by two independent observers using a four-tier scale (0 = no staining, 1 = weak, 2 = moderate, 3 = strong). The analysis focused on overall staining intensity and subcellular localization (membranous vs. cytoplasmic). Mean scores were calculated for each condition.

### 2.6. Statistics

Statistical analysis was conducted with GraphPad Prism (version 10.4.2, GraphPad Software, Boston, MA, USA). Data normality was confirmed using the Shapiro–Wilk test. For normally distributed datasets, comparisons between groups were assessed using the unpaired Student’s *t*-test; *p*-values of < 0.05 were considered statistically significant. Data are presented as the mean ± standard deviation (SD).

## 3. Results

### 3.1. Effects of the Systemic Therapeutics on SST Receptors’ Expression

**Figure 1:**
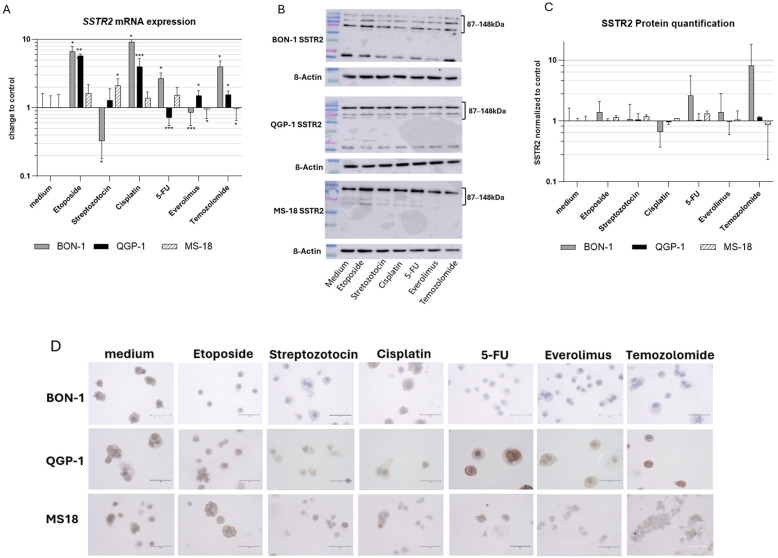
(**A**) *SSTR2* mRNA expression relative to controls. Expression normalized to the housekeeping gene GAPDH (mean ± SD; *n* = 6 experimental repeats; * *p* < 0.05, ** *p* < 0.01, *** *p* < 0.001). (**B**) Western blot of SSTR2 and β-actin as a loading control. SSTR2 appears at ~87/148 kDa, consistent with glycosylated and oligomeric receptor forms (**C**) SSTR2 protein quantification of the Western blot with the medium control (*n* = 3). (**D**) Immunohistochemical analysis of SSTR2 in BON-1, QGP-1, and MS-18 cells. Representative cytospin images show cells as single cells or small clusters. Staining ranges from absent to strong, with low-expressing conditions showing weak or diffuse signals without clear membranous localization and higher-expressing conditions showing more pronounced staining. Intensity was assessed using a four-tier scale (0–3). Scale bar: 75 µm.

The expression of SSTR2 and SSTR5 following incubation with systemic therapeutics were assessed using qRT-PCR and Western blot analysis. In addition, SSTR2 expression was further evaluated by immunohistochemistry (IHC). The results are presented in Figure 1.

### 3.2. SSTR2

Following exposure to the different systemic therapeutics, the BON-1 cell line demonstrated a significant increase in *SSTR2* mRNA expression after treatment with etoposide, cisplatin, 5-FU, and TMZ, whereas STZ and everolimus led to decreased expression. At the protein level, similar trends were observed; however, these changes did not reach statistical significance in Western blot quantification. SSTR2 was detected at ~87 and ~148 kDa, consistent with glycosylated and higher-order receptor forms. Notably, cisplatin represented an exception, showing a trend toward reduced SSTR2 protein levels.

In the QGP-1 cell line, *SSTR2* mRNA expression was significantly upregulated following treatment with etoposide, cisplatin, everolimus, and TMZ, while 5-FU induced a downregulation. STZ had no significant effect. Protein-level alterations were modest and did not reach significance but were generally consistent with the mRNA findings.

The MS-18 cell line exhibited a modest increase in *SSTR2* mRNA expression after STZ treatment, along with trends toward increased expression following etoposide, cisplatin, and 5-FU. In contrast, everolimus and TMZ induced a significant decrease, although the absolute magnitude of this effect was minimal. Protein-level changes were similarly small and did not reach statistical significance (Figure 1A–C). The full Western blots are provided in Supplemental Figure S1.

Immunohistochemical analysis showed heterogeneous SSTR2 expression across cell lines and treatment conditions (Figure 1D). MS-18 cells exhibited higher baseline staining intensity with partially membranous localization, whereas BON-1 and QGP-1 cells displayed overall weaker and more variable staining. Treatment effects were cell line-dependent without a uniform pattern. Etoposide was associated with a tendency toward increased staining, while other agents showed variable effects, including reduced staining in selected conditions. Semi-quantitative scoring (0–3) confirmed these observations, indicating only modest differences between treatments (see Supplementary Table S2). Interpretation is limited by the variability inherent to cytospin-based preparations.

### 3.3. SSTR5

In the BON-1 cell line, *SSTR5* mRNA expression was downregulated following treatment with etoposide, STZ, cisplatin, and 5-FU, whereas everolimus and TMZ induced upregulation. At the protein level, a similar decrease was observed after etoposide, STZ, and cisplatin treatment, while increased expression was detected following 5-FU, everolimus, and TMZ exposure. SSTR5 was detected as a broad band at ~50–70 kDa, exceeding the predicted molecular weight, consistent with heterogeneous glycosylation of the receptor.

In contrast, QGP-1 cells exhibited increased *SSTR5* mRNA expression after treatment with etoposide, STZ, cisplatin, and everolimus, whereas 5-FU reduced expression and TMZ showed a similar downward trend. Changes in SSTR5 protein levels were consistent with mRNA findings for 5-FU, everolimus, and TMZ, but diverged from the mRNA patterns observed after etoposide, STZ, and cisplatin treatment.

**Figure 2:**
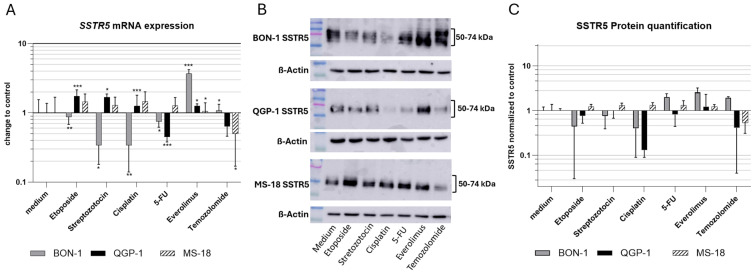
(**A**) *SSTR5* mRNA expression relative to controls. Expression normalized to the housekeeping gene GAPDH (mean ± SD; *n* = 6 experimental repeats; * *p* < 0.05, ** *p* < 0.01, *** *p* < 0.001). (**B**) Western blot of SSTR5 and β-actin as a loading control. SSTR5 (predicted size ~39 kDa; observed bands ~50–74 kDa) is known to migrate above its calculated molecular weight, consistent with glycosylation. (**C**) SSTR5 protein quantification of the Western blot with the medium control (*n* = 3).

In MS-18 cells, *SSTR5* mRNA and protein levels showed consistent trends across all treatments, with increased expression following etoposide, STZ, cisplatin, 5-FU, and everolimus, and decreased expression after TMZ. However, statistical significance was achieved only at the mRNA level, specifically after treatment with everolimus and TMZ (Figure 2). The full Western blots are provided in Supplemental Figure S2.

### 3.4. Effects on ^68^Ga-DOTATOC Uptake

In the BON-1 cell line, ^68^Ga-DOTATOC uptake increased following treatment with etoposide, cisplatin, 5-FU, and TMZ, whereas everolimus led to a decrease. STZ had no significant effect.

In QGP-1 cells, a significant increase in ^68^Ga-DOTATOC uptake was observed after etoposide treatment, with more modest induction following STZ and everolimus. In contrast, trends toward a reduced uptake were seen after cisplatin and 5-FU, while TMZ showed a tendency toward increased uptake.

**Figure 3:**
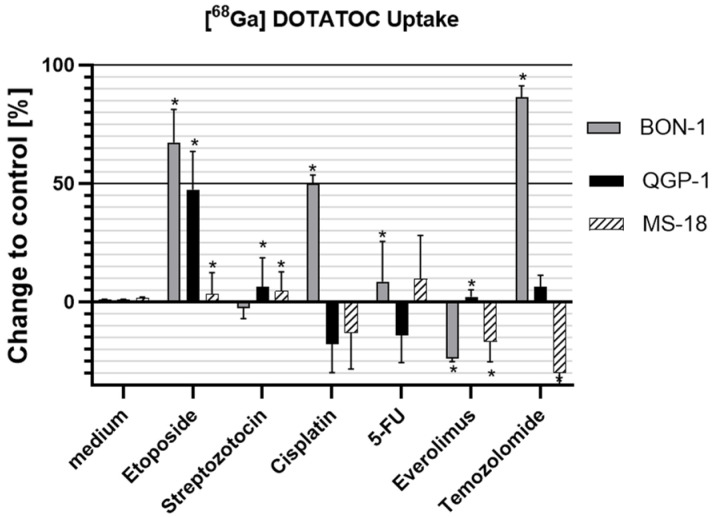
Uptake of radiolabeled ^68^Ga-DOTATOC in BON-1, QGP-1, and MS-18 cells following drug treatment. Data normalized to untreated controls (mean ± SD; *n* = 3; * *p* < 0.05).

In the MS-18 cell line, etoposide and STZ induced a significant but modest increase in ^68^Ga-DOTATOC uptake, with a similar trend observed after 5-FU exposure. Conversely, everolimus and TMZ significantly reduced uptake, and cisplatin showed a trend towards decreased uptake (Figure 3).

## 4. Discussion

To our knowledge, this study is the first to systematically evaluate how guideline-conforming systemic therapies for NENs modulate SSTR2 and SSTR5 expression, as well as radioligand uptake, in a structured, substance- and cell line-dependent manner. The present study systematically evaluated the effects of multiple chemotherapeutic and targeted agents on somatostatin receptor (SSTR) expression across three neuroendocrine neoplasm (NEN) cell lines, integrating mRNA, protein, and functional readouts. Our data demonstrate that SSTR modulation is drug-specific and strongly cell line-dependent. Notably, etoposide consistently induced *SSTR2* upregulation at the transcriptional level, which was mirrored by increased protein levels and radioligand uptake. Streptozotocin, 5-FU, and everolimus showed only minor and heterogeneous effects on receptora’ expression and activity. These findings indicate that certain therapeutic agents can modulate SSTR expression and may therefore influence the efficacy of SSTR-targeted diagnostic or therapeutic approaches.

Previous preclinical work has primarily highlighted the potential of single chemotherapeutics or epigenetic drugs to induce SSTR2 upregulation [21,25]. Our study extends this framework by systematically comparing six clinically approved systemic agents and including the primary MS-18 cell line with considerably higher baseline SSTR2 expression than the BON-1 and QGP-1 cell lines [28].

Consistent with earlier studies, we confirmed that systemic agents can modulate SSTR expression and thereby alter the efficacy of SSTR-targeted imaging and therapy [21,24]. Shah et al. (2021) [21] showed that TMZ and STZ enhanced SSTR2 expression and radionuclide uptake in BON-1 cells, with the strongest effect observed in cells with low baseline receptor density. Jin et al. (2019) [25] demonstrated SSTR2 induction by 5-FU, particularly when combined with demethylating agents—an approach currently with limited clinical applicability because epigenetic modulators are not approved for NET treatments [21,25].

Our findings confirm the observation by Shah et al. that TMZ upregulates SSTR2 in BON-1 cells, resulting in increased radionuclide uptake. However, our study expands substantially upon earlier works. We reveal that TMZ does not universally induce SSTR2 or SSTR5. In the SSTR-low BON-1 cell line, TMZ induced a significant increase in *SSTR2* and *SSTR5* expression, corresponding to an increase in ^68^Ga-DOTATOC uptake. In the SSTR-high cell line MS-18, no impact on *SSTR2* expression with a decrease in *SSTR5* was observed, resulting in a decrease in ^68^Ga-DOTATOC uptake. The significant impact on ^68^Ga-DOTATOC uptake underscores the biological relevance of the mRNA changes, while the lack of protein-level significance likely reflects post-transcriptional regulation and temporal dynamics. The overall closer correlation of ^68^Ga-DOTATOC uptake with *SSTR2* rather than *SSTR5* expression may be explained by its higher binding affinity for SSTR2 compared with SSTR5 [30].

These observations underscore a key limitation of prior studies: as NENs are a heterogeneous group of neoplasia, observations derived from a single cell line with low SSTR expression cannot be generalized. Furthermore, it can be hypothesized that in high SSTR-expressing cells, there is limited room for further upregulation. Substances that induce increased SSTR expression in cells with low receptor composition might have even a reverse effect. This hypothesis is supported by the work of Shah et al., who demonstrated negligible TMZ-induced increases in SSTR expression in a high-SSTR2 NCI-H722 line.

Incubation with etoposide induced increased the expression of *SSTR2* in all three cell lines, with similar effects on SSTR5 in QGP-1 and MS-18 cells, resulting in an increase in ^68^Ga-DOTATOC uptake in all three cell lines. The response on STZ, cisplatin, 5-FU, and everolimus showed high heterogeneity between the cell lines, resulting in a decrease in SSTR expression and ^68^Ga-DOTATOC uptake in some cell lines. In contrast to the cytotoxic therapeutics, everolimus may affect SSTR expression via mTOR-dependent regulation of protein synthesis and transcription. The measured *SSTR2* and *SSTR5* expression levels showed significant differences after some of the studied compounds in selected cell lines (e.g., cisplatin in BON-1), suggesting cell line-specific differences in the regulation of these receptors. Notably, no consistent response pattern was observed between pancreatic (BON-1, QGP-1) and rectal (MS-18) NEN models, which may argue against a purely organ-specific effect and rather support a context-dependent interpretation. The ^68^Ga-DOTATOC uptake correlated with the change in expression of SSTR2 in most cases. IHC findings were consistent with the molecular and functional data, showing only modest changes. Given the cytospin-based preparation and semi-quantitative analysis, these are considered supportive. The inverse trend observed for cisplatin may reflect stress-induced translational suppression or enhanced protein degradation, uncoupling mRNA and protein expression.

In line with Jin et al., we observed consistent SSTR2 upregulation following 5-FU treatment in the BON-1 cell line. This effect was not found in our MS-18 and QGP-1 cell lines, underlining the heterogeneity of NEN. Similarly, Jin et al. could not reproduce the strong upregulation of SSTR2 expression after 5-FU exposure as seen in BON1 cells in their remaining cell lines either. Collectively, our findings demonstrate that SSTR2 modulation is not universal and is strongly shaped by the drug mechanism, baseline receptor status, and cellular lineage. Nevertheless, our study identifies promising agents, such as etoposide or TMZ, for future synergistic approaches to improve SSTR-directed diagnostic or therapeutic strategies.

At the same time, due to the high heterogeneity of NENs, several routinely used systemic agents such as cisplatin, 5-FU, everolimus, and temozolomide may also reduce SSTR2’s availability, raising the concern that they could diminish the diagnostic performance of SSTR-based imaging and, more critically, impair the therapeutic efficacy of SSTR-directed therapies, if administered without careful consideration of the timing and tumor biology. These are also important findings to help avoid undesired SSTR downregulation by a disadvantageous combination or overlap of treatment modalities. Further in vitro and in vivo studies are required to unravel the exact underlying molecular mechanisms and to translate these findings into a clinical setting.

SSTR2 expression is regulated at the transcriptional, epigenetic, and post-translational levels and is closely linked to neuroendocrine differentiation, which may explain the context-dependent effects observed in our study [31]. The consistent upregulation following etoposide treatment may be related to activation of the DNA damage response, including p53-associated pathways [32]. While a direct mechanistic link remains speculative, stress-induced transcriptional changes may promote the expression of differentiation-associated genes such as SSTR2.

A key strength of this study is the inclusion of functional radioligand uptake assays using ^68^Ga-DOTATOC, which integrates receptor expression, function, binding affinity, and functional availability. The inclusion of the MS-18 cell line adds an important dimension: while the findings of Shah et al. were reproduced in BON-1, they did not translate to SSTR2-high models. This poses the need for further preclinical work to explore the robustness of the first positive data for the combination of TMZ and PRRT in vivo [24]. Given the clinical use of temozolomide in combination regimens such as CAPTEM, it should be noted that capecitabine is a prodrug of 5-FU included in our analysis. While such combinations with ^177^Lu-DOTATATE are increasingly investigated, the underlying effects may reflect additive cytotoxicity rather than consistent SSTR upregulation. In line with this, the heterogeneous and context-dependent effects observed in our study argue against assuming uniform receptor modulation and highlight the need for future clinical studies assessing changes in ^68^Ga-DOTATOC/-TATE uptake under chemotherapy.

Also acknowledging the intra-patient heterogeneity of SSTR expression, a homogenization of expression with an upregulation of SSTR-low portions and corresponding downregulation in SSTR-high portions could, in principle, enhance the performance of SSTR-directed therapies, as SSTR heterogeneity is associated with inferior treatment response [33]. Taken together, these results suggest that the choice and sequencing of systemic therapies can critically influence the efficacy of SSTR-targeted strategies. For planning of treatments, it should be considered that some agents may induce receptor downregulation in receptor-rich NEC or NET cells, thereby potentially reducing therapeutic efficacy. At the same time, etoposide emerges as the most robust candidate for enhancing the efficacy of SSTR-directed therapies, warranting further validation in preclinical and clinical studies.

Our study is limited by its in vitro design, which cannot fully capture the complexity of the tumor microenvironment, including stromal interactions, vascularization, and immune components. Moreover, radioligand binding was assessed using ^68^Ga-DOTATOC rather than therapeutic isotopes. While ^68^Ga-DOTATOC PET has been shown to predict PRRT response in clinical settings [34,35], further studies are required to validate whether drug-induced receptor modulation observed in vitro translates into clinically meaningful improvements in therapeutic uptake and efficacy. Future work should also clarify the molecular mechanisms underlying drug-induced SSTR2 regulation and assess whether controlled induction of receptor expression can be harnessed to optimize PRRT in patients with receptor-low or dedifferentiated NEN.

## 5. Conclusions

Etoposide emerges as the most consistent pharmacological enhancer of SSTR2 expression and radioligand uptake across multiple NET/NEC models, providing a compelling rationale for its integration into combination or sequencing concepts with PRRT. The heterogeneous and sometimes adverse effects of other systemic agents highlight the importance of drug selection and treatment timing. While strong TMZ-induced SSTR2 upregulation can be reproduced in BON-1 cells, its absence—or inversion—in cell lines with higher baseline expression underscores the limited generalizability of prior findings. Our findings might open avenues for pharmacological receptor induction as a promising strategy for improving SSTR-directed diagnostic and therapeutic strategies, particularly in tumors with low or heterogeneous SSTR2 expression.

## Data Availability

This article features the authors’ own research data, which are available from the corresponding author upon request.

## Supplementary Materials

The following supporting information can be downloaded at https://www.mdpi.com/article/10.3390/cancers18091368/s1, Table S1: Antibodies and qPCR probes used in the experiments. Figure S1: Western blot of SSTR2 and β-actin as loading control. Figure S2: Western blot of SSTR5 and β-actin as the loading control. Table S2: Semi-quantitative immunohistochemical evaluation of SSTR2 staining across cell lines and treatment conditions. Staining intensity was scored using a four-tier scale (0 = none, 1 = weak, 2 = moderate, 3 = strong) based on assessment of three cytospin areas per condition by two independent observers. Values represent mean scores.
