# Supplementary material for: Impact of Neuroendocrine Neoplasm-Specific Systemic Treatments on Somatostatin Receptors Expression and Function in Neuroendocrine Tumor Cells
Source: Cancers (Basel). 2026 Apr 25;18(9):1368. doi: 10.3390/cancers18091368 (PMC13163034; doi:10.3390/cancers18091368)

Supplementary Table S1: *Antibodies and qPCR probes used in the experiments.*

gene symbol	*western blot*	*Immunhisto-chemistry*	*TaqMan assay ID applied biosystems*
SST2	sc-365502	ab134152	Hs_00265624_s1
SST5	ab156864	ab109495	Hs_00265647_s1
GAPDH	-	-	VIC/TAMRA #4310884E
ß-Actin	sc-47778	-	VIC/TAMRA #4310881E

Suo

Supplementary Figure S1: *Western blot of SSTR2 and ß-actin as loading control.*

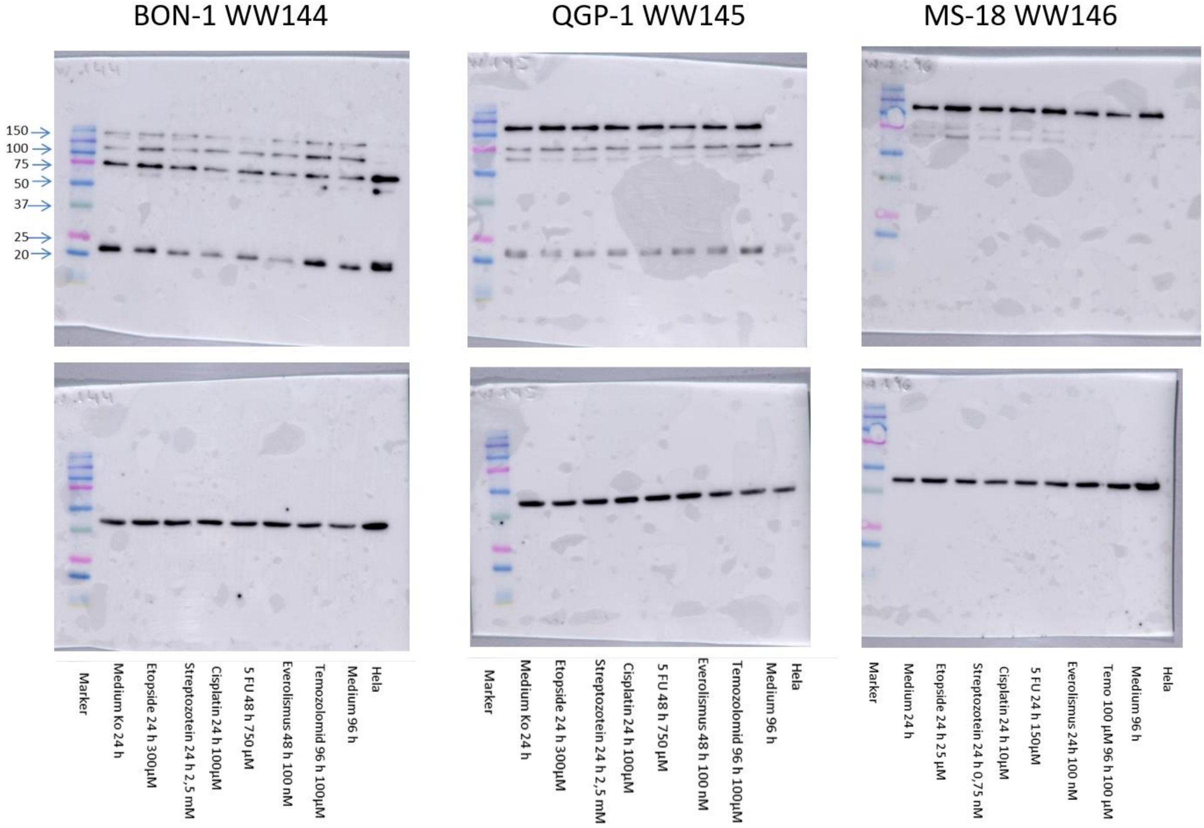

Supplementary Table S2: *Semi-quantitative immunohistochemical evaluation of SSTR2 staining across cell lines and treatment conditions. Staining intensity was scored using a four-tier scale (0 = none, 1 = weak, 2 = moderate, 3 = strong) based on assessment of three cytospin areas per condition by two independent observers. Values represent mean scores.*

	BON-1	QGP-1	MS-18
Medium	1,50	1,83	1,67
Etoposide	1,33	2,00	2,50
Streptozotocin	0,33	1,50	1,25
Cisplatin	1,33	1,00	1,50
5-FU	0,00	2,83	1,17
Everolimus	0,33	1,83	0,83
Temozolomide	0,50	2,17	1,50

Supplementary Figure S2: *Western blot of SSTR5 and ß-actin as the loading control.*

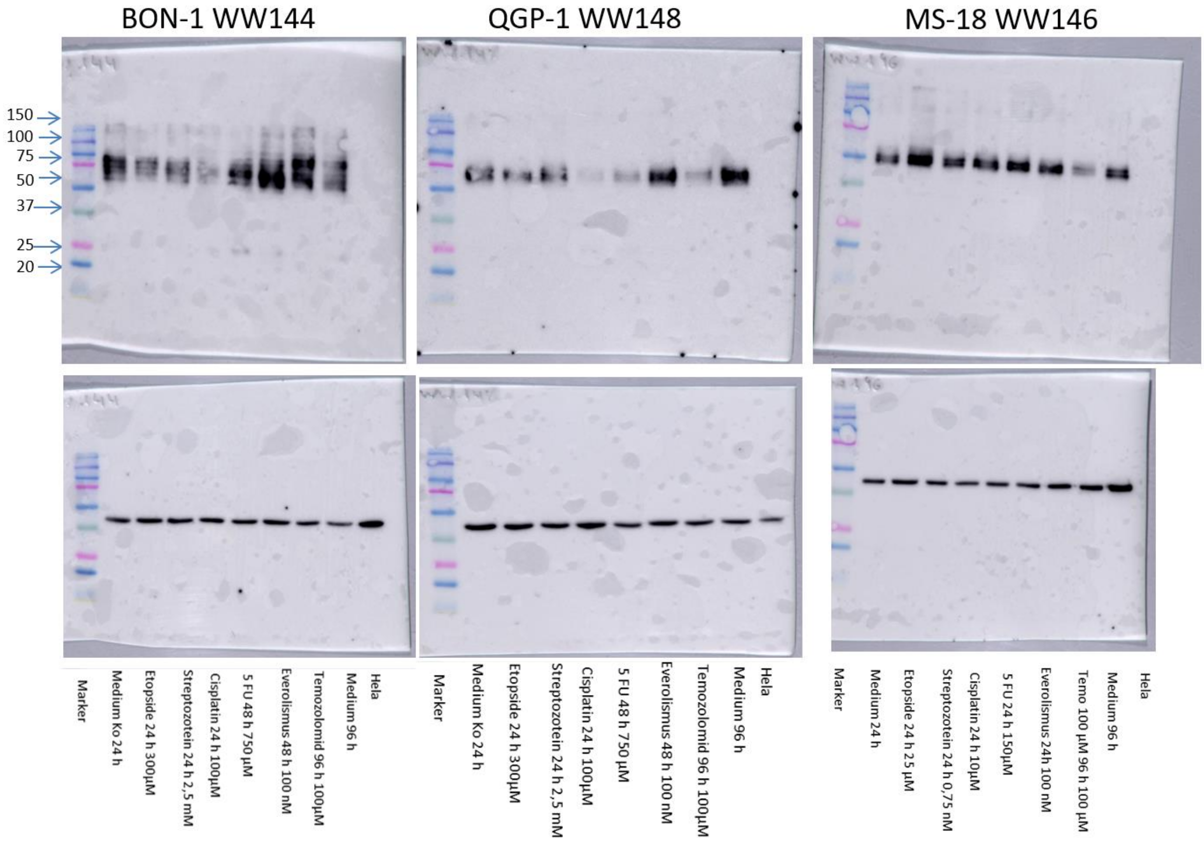

Supplement: Supplementary file 1 [file cancers-18-01368-s001.zip › cancers-4239327-supplementary.pdf]
